# A retrospective analysis of bacterial colonization epidemiology in pediatric burn patients in Qatar

**DOI:** 10.5339/qmj.2026.56

**Published:** 2026-08-25

**Authors:** Soliman Mohamed Aly, Ahmed Al Homosy, Islam Abu El Fotoh, Jimmy Thomas

**Affiliations:** 1 Pharmacy Department, Al-Wakra Hospital, Hamad Medical Corporation (HMC), Qatar

**Keywords:** Pediatric burns, burn wound colonization, antimicrobial stewardship, pseudomonas aeruginosa, wound infection, sepsis

## Abstract

**Background:**

Burn injuries in children are associated with a high risk of wound colonization and infection due to loss of skin integrity and prolonged hospitalization. Understanding temporal microbiological patterns is essential to guide clinical management and antimicrobial stewardship.

**Methods:**

A retrospective cohort study was conducted at the Pediatric Intensive Care Unit (PICU) of Al-Wakrah Hospital, Qatar, between 2022 and 2025. Children aged 1 month to 14 years with burn injuries and at least one wound culture were included. Early (≤72 hours) and late (>72 hours) wound colonization patterns, antimicrobial utilization, and sepsis outcomes were analyzed using Fisher’s exact test.

**Results:**

A total of 199 patients were included (mean age 3 years), with scald burns accounting for 86.4% and most burns involving ≤10% total body surface area (TBSA). Early wound cultures were predominantly no growth or normal flora (68.2%). In contrast, late cultures demonstrated a significant decline in normal flora (10.5% vs 27.3%, *p* < 0.001) and a shift toward Gram-negative organisms, particularly Pseudomonas aeruginosa (7.2% vs 0.5%, *p* = 0.002), along with emergence of yeast (3.9% vs 0%, *p* = 0.008). Larger burns (>10% TBSA) were significantly associated with Pseudomonas aeruginosa colonization (*p* = 0.003). Antibiotic use among patients with negative cultures increased significantly in the late phase (56.7% vs 28.8%, *p* = 0.003). Sepsis occurred in 5.5% of patients and was more frequent in larger burns, although not statistically significant.

**Conclusion:**

Pediatric burn wound microbiology demonstrates a clear temporal shift toward Gram-negative organisms with prolonged hospitalization, particularly in larger burns. Increased antibiotic use in patients with negative cultures highlights important opportunities for antimicrobial stewardship and optimization of prescribing practices.

## 1. INTRODUCTION 

Burn injuries represent a major cause of morbidity in the pediatric population worldwide, particularly among younger children.^1^ Children are at increased risk of burn-related complications due to physiological immaturity, thinner skin, and limited ability to avoid hazardous environments. Burn wounds are highly susceptible to infection because of loss of skin integrity, immune dysregulation, prolonged hospitalization, and frequent exposure to invasive procedures.^2^

Bacterial colonization of burn wounds typically begins within 48 hours of injury, originating from microorganisms residing in sweat glands and hair follicles.^3^ Gram-positive organisms, particularly Staphylococcus aureus derived from the patient’s skin and nasal flora, are usually the initial predominant colonizers. As the wound environment evolves and hospitalization is prolonged, Gram-negative organisms, such as Pseudomonas aeruginosa and Klebsiella species, increasingly emerge, especially in healthcare settings where nosocomial transmission is common.^4,5^ In patients with extensive burns, bacterial translocation from the gastrointestinal tract may also contribute to wound contamination and systemic infection.

Appropriate antimicrobial therapy plays a critical role in determining outcomes in life-threatening infections. Delays in initiating effective antimicrobial treatment are associated with increased mortality, prolonged hospital stay, and worse clinical outcomes.^6^ Conversely, inappropriate or excessive antibiotic use contributes to antimicrobial resistance and unnecessary healthcare costs.^7,8^ The Surviving Sepsis Campaign recommends administration of broad-spectrum antibiotics within one hour of recognition of severe sepsis or septic shock.^8^ Infection is also a major cause of skin graft failure in burn patients, most commonly due to pathogenic Gram-negative bacteria.^9^ Skin graft loss significantly increases morbidity and mortality through prolonged immobilization, extended hospitalization, loss of donor sites, and increased risk of sepsis. However, it is important to distinguish between wound colonization and true infection, as reliance on microbiological findings alone without clinical correlation may lead to unnecessary antibiotic use.

Prophylactic antibiotics have been shown to reduce bacterial burden in the wound bed, lower graft failure rates, shorten hospital stay, and decrease donor site infections.^10–13^ However, indiscriminate use of prophylactic antibiotics increases the risk of multidrug-resistant organisms and should be avoided. Antibiotic selection should be guided by the most likely pathogens and local epidemiological patterns.^14^

Scald burns represent the most common type of burn injury in children, with the upper limbs most frequently affected, followed by the lower limbs and torso.^15^ The severity of infection is strongly associated with burn size, particularly in patients with burns exceeding 40% of the total body surface area (TBSA), where up to 75% of deaths are attributable to sepsis or infection-related complications.^16,17^

Despite growing literature on burn wound microbiology, there is limited region-specific data from the Middle East, particularly in pediatric populations. Furthermore, few studies have evaluated temporal changes in colonization patterns alongside antimicrobial prescribing practices. Therefore, this study aims to address these gaps by providing a comprehensive evaluation of early and late wound colonization patterns, antimicrobial utilization, and antimicrobial susceptibility profiles.

The primary objective of this study was to characterize early (≤72 hours) and late (>72 hours) wound microbiological patterns in pediatric burn patients admitted to the Pediatric Intensive Care Unit (PICU) at Al-Wakrah Hospital, the national burn center in Qatar. Secondary objectives are to evaluate antimicrobial prescribing practices, assess antimicrobial susceptibility profiles, and identify factors associated with infectious complications such as sepsis.

## 2. METHODS

### 2.1. Study design and setting

A retrospective observational cohort study was conducted over a three-year period from January 2022 to March 2025 at the PICU of Al-Wakrah Hospital, Qatar. Al-Wakrah Hospital serves as the national burn center and receives pediatric burn patients from across the country.

The study was designed to evaluate wound bacterial colonization patterns, antimicrobial prescribing practices, antimicrobial susceptibility profiles, and infectious complications among pediatric burn patients during hospitalization.

### 2.2. Study population

Children aged 1 month to 14 years admitted to the PICU with burn injuries and at least one wound culture obtained during admission were eligible for inclusion. Burn mechanisms included scald, contact, flame, chemical, and electrical burns, regardless of burn surface area. Patients admitted exclusively for post-burn scar management or reconstructive procedures without active burn wounds were excluded.

### 2.3. Data collection

Demographic and clinical data were obtained from the burn unit database and electronic medical records. Collected variables included age, sex, burn mechanism, burn depth, TBSA, microbiological findings, antimicrobial use, antimicrobial susceptibility results, and infectious outcomes. Medical records were reviewed in detail to ensure completeness and accuracy of data.

All patient data were de-identified prior to analysis. Ethical approval was obtained from the Medical Research Center (MRC) Ethics Review Committee (Approval ID: MRC-01-25-480). Due to the retrospective nature of the study, the requirement for informed consent was waived.

### 2.4. Burn characteristics and definitions

Burn surface area was estimated using the Lund and Browder chart.^18^ Severe burn injury in pediatric patients was defined as involvement of ≥10% TBSA or burns affecting critical anatomical areas, including the face, hands, feet, or genitalia.^19^

Burn depth was categorized as superficial, partial-thickness, or full-thickness. In instances of mixed burn depth and pathophysiology, the most severe burn depth was prioritized for assessment. 

### 2.5. Microbiological sampling and classification

Wound swab cultures were obtained in the Emergency Department, PICU, or operating theatre prior to wound dressing or surgical debridement. All microbiological analyses were performed by the Microbiology Department of Al-Wakrah Hospital using standard laboratory protocols. Microorganisms were categorized as Gram-positive bacteria, Gram-negative bacteria, or fungi.

To maintain data integrity, duplicate wound cultures isolating the same microorganism from the same body location within a 48-hour interval were excluded. Cultures obtained beyond this interval were included in the analysis. When different microorganisms or the same organism with different antimicrobial resistance profiles were isolated, each isolate was included as a separate observation.

### 2.6. Early and late colonization definitions

Early colonization was defined as wound cultures obtained within the first 72 hours of hospital admission. Late colonization was defined as cultures obtained beyond 72 hours after admission. These definitions were used to assess temporal changes in wound microbiology during hospitalization.^20^

### 2.7. Definitions of infection and sepsis

Burn wound infection was defined by the presence of clinical signs of infection (erythema, swelling, tenderness, purulent discharge, offensive odor, increased pain, or fever) in conjunction with a positive wound culture.^4^ Polymicrobial infection was defined as isolation of more than one pathogenic organism from a single wound sample, underscoring the complexity of infection management in burn patients. Sepsis was identified based on documented clinical diagnosis supported by systemic signs of infection and microbiological evidence, as recorded in the medical records.

### 2.8. Antibiotic use classification

Antibiotic therapy was classified into three categories:

- **Prophylactic antibiotics:** systemic antibiotics administered in the absence of documented infection or clinical signs of infection.^21^
- **Empiric antibiotics:** antibiotics initiated prior to availability of culture results in response to clinical suspicion of infection, sepsis, or patient deterioration.
- **Targeted antibiotics:** antibiotics prescribed based on wound culture results and antimicrobial susceptibility testing.

### 2.9. Infection control measures

All patients were managed in isolation rooms within the PICU. Standard infection prevention and control measures were strictly followed, including hand hygiene, use of personal protective equipment, and environmental cleaning, to minimize cross-transmission of microorganisms.

### 2.10. Outcomes

The primary outcome of the study was the prevalence and distribution of bacterial colonization in pediatric burn wound cultures during early (≤72 hours) and late (>72 hours) hospitalization periods.

Secondary outcomes included:

- Association between burn surface area (≤10% vs >10% TBSA) and wound microbiological profile.
- Age-based stratification of wound colonization patterns (≤2 years vs >2 years).
- Antibiotic utilization patterns (timing and indication).
- Occurrence of infectious complications, including sepsis.

### 2.11. Statistical analysis

The primary outcome of the study was the prevalence and distribution of bacterial colonization in pediatric burn wound cultures during early (<72 hours) and late (>72 hours) hospitalization periods. Secondary outcomes included the association between burn surface area and wound microbiological profile, antibiotic utilization patterns, and the occurrence of infectious complications.

Categorical variables were summarized as frequencies and percentages, while continuous variables were expressed as means or medians as appropriate. Comparisons of bacterial isolates between early and late periods, as well as between burn size categories (≤10% vs >10% TBSA), were performed using Fisher’s exact test due to small, expected cell counts. Risk differences with 95% confidence intervals were calculated to quantify the magnitude and direction of differences in organism prevalence and antibiotic use between groups. A two-sided p-value <0.05 was considered statistically significant. All analyses were performed using standard biostatistical methods.

This analysis was limited to using aggregated microbiological data, which precluded patient-level multivariable logistic regression and adjustment for potential confounders. Additionally, small subgroup sizes may have reduced the statistical power to detect significant associations.

## 3. RESULTS

### 3.1. Study population and demographics

A total of 199 pediatric burn patients were included. Age distribution was: <1 year 35 (17.6%), 1–2 years 100 (50.3%), 3–6 years 40 (20.1%), 7–10 years 16 (8.0%), and 11–14 years 8 (4.0%). Scald burns were reported in 172 patients (86.4%), thermal burns in 19 (9.5%), friction burns in 5 (2.5%), chemical burns in 2 (1.0%), and electrical burns in 1 (0.5%). TBSA involvement was <5% in 55 patients (27.6%), 5–10% in 107 (53.8%), and >10% in 37 (18.6%) (Table 1).

### 3.2. Wound colonization patterns: Early versus late hospitalization

A total of 183 early cultures (≤72 hours) and 152 late cultures (>72 hours) were analyzed. No growth was reported in 75 (41.0%) early cultures and 83 (54.6%) late cultures. Normal skin flora was reported in 50 (27.3%) early cultures and 16 (10.5%) late cultures. Methicillin-sensitive Staphylococcus aureus (MSSA) was identified in 30 (16.4%) early cultures and 18 (11.8%) late cultures. Methicillin-resistant Staphylococcus aureus (MRSA) was identified in 17 (9.3%) early cultures and 8 (5.3%) late cultures. Pseudomonas aeruginosa was identified in 1 (0.5%) early culture and 11 (7.2%) late cultures. Yeast was identified in 6 (3.9%) late cultures and not identified in early cultures (Table 2).

### 3.3. Wound colonization by burn size

Among patients with TBSA ≤10%, no growth was reported in 113 (56.5%) and normal flora in 46 (23.0%). Among patients with TBSA >10%, no growth was reported in 45 (48.9%) and normal flora in 20 (21.7%). MSSA was identified in 35 (17.3%) patients with ≤10% TBSA and 6 (6.5%) patients with >10% TBSA. MRSA was identified in 21 (10.4%) and 4 (4.3%), respectively. Pseudomonas aeruginosa was identified in 3 (1.6%) patients with ≤10% TBSA and 8 (8.7%) patients with >10% TBSA (Table 3).

### 3.4. Age-stratified analysis of wound colonization

A total of 213 cultures were obtained from patients aged ≤2 years and 115 cultures from patients aged >2 years. No growth or normal flora was reported in 142 (66.7%) cultures in patients aged ≤2 years and 75 (65.2%) in patients aged >2 years. MSSA was identified in 37 (17.4%) and 11 (9.6%) cultures, respectively. MRSA was identified in 15 (7.0%) and 10 (9.6%). Pseudomonas aeruginosa was identified in 8 (3.8%) and 4 (4.3%). Other organisms were identified in 11 (5.2%) and 13 (11.3%) (Table 4).

### 3.5. Antibiotic use in patients with no growth or normal flora

Antibiotics were prescribed in 34 of 118 patients (28.8%) in the early period and 17 of 30 patients (56.7%) in the late period (Table 5).

### 3.6. Antibiotic selection and timing

A total of 147 antibiotic prescriptions were analyzed: 74 early and 73 late. Frequency is presented in Table 6.

### 3.7. Antimicrobial susceptibility patterns

Susceptibility results are presented in Tables 7 and 8.

### 3.8. Sepsis outcomes

Sepsis occurred in 11 patients. Among patients with TBSA ≤10%, sepsis occurred in 7 (4.3%). Among patients with TBSA >10%, sepsis occurred in 4 (10.8%) (Table 9). Among patients aged ≤2 years, sepsis occurred in 8 (5.9%). Among patients aged >2 years, sepsis occurred in 3 (4.7%) (Table 10). Distribution of organisms among sepsis and non-sepsis cases is presented in Table 11.

## 4. DISCUSSION

This study evaluated wound colonization patterns, antibiotic use, and sepsis occurrence among pediatric burn patients, stratified by age, burn size, and timing of culture acquisition. The findings demonstrate variation in microbiological profiles over time, differences according to burn size, and frequent antibiotic use in the absence of microbiological confirmation of infection.

Age-based analysis showed comparable proportions of cultures yielding no growth or normal skin flora between younger (≤2 years) and older children. Although MSSA was more frequently isolated in younger children and other organisms were more frequently identified in older children, these differences were not statistically significant for most organisms. Sepsis incidence was also similar between age groups. These findings may suggest that age-related differences in immune maturity influence colonization patterns without clearly affecting progression to systemic infection within this cohort. This observation is consistent with previous reports indicating that colonization does not necessarily predict invasive infection in pediatric burn patients.^22,23^

Burn size demonstrated differences in both microbiological findings and clinical outcomes. Patients with burns involving >10% TBSA had a higher proportion of Pseudomonas aeruginosa isolates and a higher numerical rate of sepsis compared with those with smaller burns, although this difference did not reach statistical significance. The increased presence of Gram-negative organisms in larger burns may be related to greater disruption of the skin barrier and increased exposure during hospitalization. These findings are consistent with published literature describing an association between burn severity and shifts in microbial colonization and infection risk.^24–26^

It is also important to distinguish between wound colonization and wound infection. The presence of microorganisms in wound cultures does not necessarily indicate invasive infection, and clinical assessment remains essential when interpreting microbiological findings and guiding treatment decisions.

Analysis of wound cultures according to timing demonstrated that no growth and normal skin flora were the most frequent findings, particularly in early cultures obtained within 72 hours of admission. In later cultures, there was an increase in no-growth results and a reduction in normal flora, alongside the emergence of organisms such as Pseudomonas aeruginosa and yeast. This temporal pattern may reflect changes in the wound environment and hospital exposure over time. The predominance of minimal or absent growth in early cultures is consistent with previous studies reporting limited colonization immediately following burn injury, with pathogenic organisms emerging later during hospitalization.^22,24^

The distribution of bacterial isolates in this cohort, including Staphylococcus aureus and Pseudomonas aeruginosa, is consistent with patterns reported in pediatric burn literature. A temporal shift toward Gram-negative organisms with prolonged hospitalization has also been described in previous studies.^24^ The relatively low prevalence of multidrug-resistant organisms in this cohort may reflect local infection control practices and antibiotic policies, although this should be interpreted cautiously.

Antibiotic use was observed in patients with wound cultures demonstrating no growth or normal skin flora. In the early period, antibiotics were prescribed in 28.8% of cases, increasing to 56.7% in the late period. In addition, the pattern of antibiotic use shifted from predominantly empiric in the early phase to predominantly prophylactic in the later phase. These findings indicate that antibiotic exposure increased over time despite the absence of microbiological evidence of infection.

Several factors may explain this pattern, including clinical concern for infection progression, difficulty distinguishing colonization from early infection, and variability in prescribing practices. However, the observed increase in antibiotic use in patients without microbiological confirmation highlights a potential area for antimicrobial stewardship interventions. From a quantitative perspective, the increase from 28.8% to 56.7% represents a substantial rise in antibiotic exposure in the setting of negative cultures. Previous studies and systematic reviews have reported that antimicrobial overuse in burn patients does not necessarily reduce infection rates and may contribute to antimicrobial resistance and adverse outcomes.^25^ These findings are consistent with the need for more targeted antibiotic strategies in pediatric burn care.

Antimicrobial susceptibility patterns in this cohort demonstrated preserved activity of commonly used agents against both Gram-positive and Gram-negative organisms. MSSA isolates showed full susceptibility to first-line agents, while MRSA remained susceptible to vancomycin. Gram-negative organisms demonstrated susceptibility to cefepime and carbapenems. These findings are broadly consistent with previously reported susceptibility patterns in similar settings, although interpretation should be made cautiously in the absence of longitudinal resistance data.

Sepsis occurred in a small proportion of patients, with higher numerical rates observed in patients with larger burns and in younger children; however, these differences were not statistically significant. The limited number of sepsis events restricts the ability to draw definitive conclusions regarding risk factors. Similarly, no clear association was observed between specific organisms and sepsis occurrence.

This study has several limitations. The retrospective design and the relatively small number of sepsis events limit statistical power and the ability to detect significant associations. In addition, key clinical variables, including length of hospital stay, intensive care unit admission and duration, use of invasive devices, and mortality outcomes, were not available. The absence of these variables limits comprehensive assessment of infection risk and antimicrobial use. Furthermore, multivariable analysis was not performed, which restricts adjustment for potential confounders. The study is also subject to selection bias, as only patients with available wound cultures were included.

Despite these limitations, the study provides detailed characterization of wound microbiology and antibiotic use patterns in pediatric burn patients. The findings highlight variation in colonization over time and identify potential discrepancies between microbiological findings and antibiotic prescribing practices, supporting the importance of antimicrobial stewardship in this population.

## 5. CONCLUSION

In this pediatric burn cohort, wound colonization demonstrated a temporal shift from normal skin flora in the early post-burn period toward Gram-negative organisms, particularly Pseudomonas aeruginosa, with prolonged hospitalization. Burn size was a key determinant of microbial patterns, with larger burns associated with higher risk of Gram-negative colonization and sepsis. Empiric antibiotic therapy was appropriately concentrated in the early phase, transitioning to targeted treatment guided by culture results. The observed antimicrobial susceptibility patterns support current treatment practices while highlighting the need for ongoing surveillance and antimicrobial stewardship. These findings provide clinically relevant evidence to guide infection prevention and antimicrobial strategies in pediatric burn care.

## ACKNOWLEDGEMENT

The author(s) have no acknowledgements to declare.

## AUTHORS’ CONTRIBUTION

SA conceived and designed the study, performed the data analysis, and prepared the initial manuscript draft. AA contributed to investigation and data collection. IA contributed to investigation and visualization. JT provided supervision. All authors contributed to the critical review and editing of the manuscript, approved the final version, and agreed to be accountable for all aspects of the work.

## CONFLICT OF INTEREST

The authors have no conflicts of interest to declare.

## DISCLOSURE OF AI USE

The author acknowledge that ChatGPT (OpenAI) tool was used solely to assist the authors with language editing, clarity, and refinement of the manuscript text. The tool was not used for study design, data collection, statistical analysis, interpretation of results, or generation of scientific findings. All scientific content, analyses, interpretations, and final decisions were reviewed and verified by the authors, who take full responsibility for the content of the manuscript.

## DATA AVAILABILITY

The data supporting the findings of this study are not publicly available due to patient confidentiality and institutional policies of Hamad Medical Corporation (HMC). Data may be made available from the corresponding author upon reasonable request, subject to HMC policies, ethical requirements, and appropriate institutional approval.

## FUNDING

The study was not funded by any organization.

## ETHICAL APPROVAL

Ethical approval was obtained from the Medical Research Center (MRC) Ethics Review Committee, Qatar, under approval number MRC-01-25-480. The requirement for informed consent was waived because of the retrospective nature of the study.

## Figures and Tables

**Table 1.** Demographic and burn-related characteristics of pediatric burn patients.

Characteristic	*n* (%)
**Age (years)**	
<1 year	35 (17.6)
1–2 years	100 (50.3)
3–6 years	40 (20.1)
7–10 years	16 (8.0)
11–14 years	8 (4.0)
**Type of burn**	
Scald	172 (86.4)
Thermal (flame/contact)	19 (9.5)
Friction	5 (2.5)
Chemical	2 (1.0)
Electrical	1 (0.5)
**TBSA**	
<5%	55 (27.6)
5–10%	107 (53.8)
>10%	37 (18.6)

**Table 2.** Distribution of bacterial species isolated from pediatric burn wounds in early (≤72 h) and late (>72 h) periods of admission.

Organism	Early *n* (%) (*n* = 183)	Late *n* (%) (*n* = 152)	*p*-value
No growth	75 (41.0)	83 (54.6)	0.016
Normal flora	50 (27.3)	16 (10.5)	<0.001
MSSA	30 (16.4)	18 (11.8)	0.28
MRSA	17 (9.3)	8 (5.3)	0.21
Mixed growth	5 (2.7)	3 (2.0)	0.74
Pseudomonas	1 (0.5)	11 (7.2)	0.002
Enterobacter	1 (0.5)	2 (1.3)	0.60
Klebsiella	0 (0.0)	3 (2.0)	0.09
E. coli	0 (0.0)	2 (1.3)	0.46
Yeast	0 (0.0)	6 (3.9)	0.008

**Table 3.** Wound culture isolates by burn size.

Organism	≤10% TBSA *n* (%)	>10% TBSA *n* (%)	*p*-value
No growth	113 (56.5)	45 (48.9)	0.24
Normal flora	46 (23.0)	20 (21.7)	0.82
MSSA	35 (17.3)	6 (6.5)	0.018
MRSA	21 (10.4)	4 (4.3)	0.11
Pseudomonas aeruginosa	3 (1.6)	8 (8.7)	0.003
Yeast	4 (2.0)	2 (2.2)	0.92

**Table 4.** Wound culture isolates by age group.

Organism	≤2 years (*n* = 213 cultures) *n* (%)	>2 years (*n* = 115 cultures) *n* (%)	*p*-value
No growth / Normal skin flora	142 (66.7)	75 (65.2)	0.81
MSSA	37 (17.4)	11 (9.6)	0.07
MRSA	15 (7.0)	10 (9.6)	0.52
Pseudomonas aeruginosa	8 (3.8)	4 (4.3)	0.77
Other organisms^†^	11 (5.2)	13 (11.3)	0.048

† Other organisms include mixed growth, Enterobacter, Klebsiella, Escherichia coli, and yeast. This classification is based on the organisms listed individually in Table 2.

**Table 5.** Antibiotic use among pediatric burn patients with no growth or normal skin flora on wound culture.

	Total patients	Received antibiotics *n* (%)	Empiric *n* (%)	Prophylactic *n* (%)	*p*-value
Early (<72 h)	118	34 (28.8)	28 (82.4)	6 (17.6)	
Late (>72 h)	30	17 (56.7)	5 (29.4)	12 (70.6)	0.003

**Table 6.** Comparison of antibiotic prescribing patterns in pediatric burn patients during early (<72 h) and late (>72 h) hospitalization periods.

Antibiotic	Early (<72 h) *n* (%)	Late (>72 h) *n* (%)	Total *n* (%)	*p*-value
Amoxicillin/clavulanate	27 (36.5%)	13 (17.8%)	40 (27.2%)	0.012
Clindamycin	15 (20.3%)	19 (26.0%)	34 (23.1%)	0.30
Cloxacillin	4 (5.4%)	19 (26.0%)	23 (15.6%)	0.0001
Ceftriaxone	19 (25.7%)	3 (4.1%)	22 (15.0%)	0.0002
Vancomycin	4 (5.4%)	5 (6.8%)	9 (6.1%)	0.74
Piperacillin/tazobactam	2 (2.7%)	4 (5.5%)	6 (4.1%)	0.44
Cefazoline	2 (2.7%)	3 (4.1%)	5 (3.4%)	0.65
Cefepime	1(1.4%)	2 (2.7%)	3 (2.0%)	0.61
Cotrimoxazole	0 (0%)	4 (5.5%)	4 (2.7%)	0.12
Meropenem	0 (0%)	1 (1.4%)	1 (0.7%)	1.0

**Table 7.** Antimicrobial susceptibility profile of Gram-negative isolates from pediatric burn wound cultures.

Organism	Gentamicin %	Cefepime %	Piperacillin-Tazobactam %	Ceftriaxone %	Ertapenem %	Meropenem %
Pseudomonas aeruginosa (*n* = 12)	83	92	83	–	–	–
Enterobacter cloacae (*n* = 3)	100	100	–	0	100	100
Klebsiella spp. (*n* = 3)	100	100	–	100	100	100
E. coli (*n* = 2)	50	50	–	50	100	100

**Table 8.** Antimicrobial susceptibility profile of Gram-positive isolates from pediatric burn wound cultures.

Organism (*n*)	Cefazolin	Cloxacillin	Gentamicin	Sulfa-methoxazole/Trimethoprim	Clinda-mycin	Vanco-mycin	Penicillin
MSSA (*n* = 48)	48 (100%)	48 (100%)	45 (93.8%)	Not tested	46 (95.8%)	Not tested	–
MRSA (*n* = 25)	0 (0%)	0 (0%)	17 (68%)	22 (88%)	20 (80%)	25 (100%)	–
Streptococcus anginosus (*n* = 1)	Not tested	Not tested	Not tested	Not tested	Not tested	Not tested	1 (100%)
Streptococcus agalactiae (*n* = 1)	Not tested	Not tested	Not tested	Not tested	Not tested	Not tested	1 (100%)
Streptococcus pyogenes (*n* = 2)	Not tested	Not tested	Not tested	Not tested	Not tested	Not tested	2 (100%)

**Table 9.** Sepsis occurrence according to burn size in pediatric burn patients.

Burn size	Sepsis *n* (%)	No sepsis *n* (%)	*p*-value
≤10% TBSA	7 (4.3)	156 (95.7)	0.30
>10% TBSA	4 (10.8)	33 (89.2)	

**Table 10.** Sepsis occurrence according to age in pediatric burn patients.

Age	Sepsis *n* (%)	No sepsis *n* (%)	*p*-value
≤2 years (*n* = 135)	8 (5.9%)	127 (94.1%)	
>2 years (*n* = 135)	3 (4.7%)	61 (95.3%)	0.74

**Table 11.** Association between wound culture organisms and sepsis.

Organism	Sepsis (*n*)	No Sepsis (*n*)	OR
Pseudomonas aeruginosa	2	10	0.68
Methicillin-resistant Staphylococcus aureus	5	20	1.26
Methicillin-sensitive Staphylococcus aureus	3	45	0.17
Streptococcus pyogenes	1	1	Unstable
